# The translation of sports injury prevention and safety promotion knowledge: insights from key intermediary organisations

**DOI:** 10.1186/s12961-017-0189-5

**Published:** 2017-03-28

**Authors:** Sheree Bekker, Penny Paliadelis, Caroline F. Finch

**Affiliations:** 1 Australian Collaboration for Research into Injury in Sport and its Prevention (ACRISP), Federation University Australia, PO Box 663, Ballarat, Victoria Australia; 2 Faculty of Health, Federation University Australia, PO Box 663, Ballarat, Victoria 3353 Australia

**Keywords:** Knowledge translation, Dissemination, Implementation, Injury prevention, Safety promotion, Sport

## Abstract

**Background:**

A recognised research-to-practice gap exists in the health research field of sports injury prevention and safety promotion. There is a need for improved insight into increasing the relevancy, accessibility and legitimacy of injury prevention and safety promotion research knowledge for sport settings. The role of key organisations as intermediaries in the process of health knowledge translation for sports settings remains under-explored, and this paper aims to determine, and describe, the processes of knowledge translation undertaken by a set of key organisations in developing and distributing injury prevention and safety promotion resources.

**Methods:**

The National Guidance for Australian Football Partnerships and Safety (NoGAPS) project provided the context for this study. Representatives from five key NoGAPS organisations participated in individual face-to-face interviews about organisational processes of knowledge translation. A qualitative descriptive methodology was used to analyse participants’ descriptions of knowledge translation activities undertaken at their respective organisations.

**Results:**

Several themes emerged around health knowledge translation processes and considerations, including (1) identifying a need for knowledge translation, (2) developing and disseminating resources, and (3) barriers and enablers to knowledge translation.

**Conclusions:**

This study provides insight into the processes that key organisations employ when developing and disseminating injury prevention and safety promotion resources within sport settings. The relevancy, accessibility and legitimacy of health research knowledge is foregrounded, with a view to increasing the influence of research on the development of health-related resources suitable for community sport settings.

**Electronic supplementary material:**

The online version of this article (doi:10.1186/s12961-017-0189-5) contains supplementary material, which is available to authorized users.

## Background

In healthcare research, the time lag between evidence being produced and its use in practice is an average of 17 years [1]. Knowledge translation has emerged as an important research area concerned with reducing this lag by determining how research findings can best inform guidelines, policy and practice [2]. WHO ([3], p. 2) defines knowledge translation as “*the synthesis, exchange and application of knowledge by relevant stakeholders to accelerate the benefits of global and local innovation in strengthening health systems and advancing people’s health*”. Key functions of knowledge translation include addressing the relevancy (timeliness, salience, actionability), accessibility (formatting and availability) and legitimacy (credibility) of research for end-users [4].

The refrain – ‘bridge the gap’ – is often used in relation to knowledge translation in health services research; however, it applies equally to any research that hopes to influence health behaviours, including sports injury prevention and safety promotion. As the phrase suggests, there exists a recognised ‘gap’ between research and practice in the field of sports injury prevention and safety promotion [5], indicated by the lack of dissemination and implementation of evidence-based interventions [6]. Implementation and dissemination science has thus emerged as a means of ‘bridging’ the efficacy to effectiveness gap in this field [6, 7]. Frameworks for this purpose have been developed for the sports injury prevention context, including the Translating Research into Injury Prevention Practice framework [8] and the Knowledge Translation Scheme [9].

The field of sports injury prevention and safety promotion has thus started to embrace the importance of knowledge translation, via implementation and dissemination research [10], as a means of addressing the relevancy, accessibility and legitimacy of research knowledge for end-users. However, knowledge translation is still often – but not always – left as recommendations for future research activities. The researchers themselves rarely provide direct safety resources or guidance for the general public based on their research, and also do not share their lessons learned from the process of dissemination. This perpetuates the gap between research and practice. One reason for this could be because the translation of research findings into practice is time-consuming and complex, and requires an understanding of the process by which research findings might influence future behaviours [2].

Key organisations involved in sport settings (government and non-government alike, such as sports governing bodies) are therefore often required to take up an intermediary role to assist in ‘bridging the gap’ by providing research-based safety knowledge to the general public in accessible forms. In this capacity, such organisations perform a knowledge translation role to inform end-users of the findings of injury prevention and safety promotion research by developing and disseminating resources [11], to hopefully positively influence the practice of safety in sport. To date, no studies in the peer-reviewed literature have identified and explained the decisions and processes that facilitate this role in the sporting context.

The aim of this study was to determine, and describe, the processes of knowledge translation undertaken by a set of key organisations in developing and distributing injury prevention and safety promotion resources. This study thus sought to provide novel insight into the knowledge translation activities undertaken by intermediary organisations that work to ‘bridge the gap’ between research and practice in sport settings. Representatives from five key intermediary organisations participated in individual face-to-face interviews, and a qualitative descriptive methodology [12] was used to understand their perceptions and experiences of the sports injury prevention and safety promotion knowledge translation role undertaken by their respective organisation.

## Methods

### Context and setting

This study was designed under the banner of the National Guidance for Australian Football Partnerships and Safety (NoGAPS) project [13], and informed by its assumptions and evolution [14]. The NoGAPS partnership organisations were the (1) Australian Football League, (2) Victorian Health Promotion Foundation, (3) New South Wales Sporting Injuries Committee, (4) JLT Sport as a division of Jardine Lloyd Thompson Australia Pty Ltd., (5) Sport and Recreation Victoria, and (6) Sports Medicine Australia. The main aim of the overarching NoGAPS project was to identify the factors that influence the translation of safety promotion interventions into practice in community sport, particularly Australian football. The original partnership goals were therefore to reduce gaps between (1) policy and practice, (2) efficacy to effectiveness, (3) research knowledge to translation, and (4) elite and community sport settings [13].

These organisations were chosen for the original NoGAPS project because they are recognised as key stakeholders in safety promotion in Australia, especially as it applies to the sport of Australian football. This group is representative of organisations at both the national and state levels concerned with sports safety promotion in Australia. Whilst the larger NoGAPS project focused on Australian football, the work undertaken at the majority of the organisations included in this partnership is not limited to Australian football alone. Therefore, while the outcomes arising from this research will be weighted towards the Australian football setting, they should resonate with a wider range of Australian and similar international community sport settings.

### Ethical approval

Ethical approval was obtained from the Federation University Australia Human Research Ethics Committee (approval number E13-015).

### Recruitment of participants

The NoGAPS partnership project provided a clear and purposeful sampling of the types of organisations that this study aimed to include. The participants in this study were the self-nominated representatives of the NoGAPS partnership project organisations – as per the original NoGAPS project [14]. The representatives were initially informed of this particular study at a face-to-face NoGAPS whole-of-partnership management meeting in 2013, at which the lead researcher presented this proposed study. A formal invitation to participate was thereafter sent to each representative via email. Six representatives from five organisations agreed to participate (one organisation provided two representatives; participants denoted as 1a, 1b, 2, 3, 4 and 5 in the results section). The sixth organisation’s representative declined to participate due to time constraints.

### Data collection

Face-to-face semi-structured interviews were conducted between July and September 2014. The interview format ensured flexibility to explore processes at the level of each organisation, as it was assumed that there would be variation across NoGAPS organisations with regards to adopted processes, and between participants with regards to the information they had and were able to disclose.

A plain language statement, informed consent form and interview schedule were sent to the participants two days prior to the scheduled interview time. Interviews were conducted at the Melbourne, Australia, offices of each NoGAPS organisation, at a date and time mutually convenient to the participant (NoGAPS representative) and interviewer (lead author). At the interviews, participants were provided with hardcopies of the plain language statement, informed consent form and interview schedule. The informed consent form was signed prior to commencement of the interview. Each interview lasted between 30 and 45 minutes, was semi-structured, and comprised open-ended questions and prompts (Additional file 1: Appendix A). The interviews were recorded using a password-protected iPhone and iPad(™).

### Organisation and preparation of data

The interviews were transcribed by a professional transcription company. Each participant was provided with the transcript of their interview for perusal, clarification and approval before the transcript was de-identified by removing participant and organisational names, and any identifying phrases. The transcripts were imported into NVivo qualitative data analysis software (QSR International Pty Ltd. V.10, 2012) as separate document sources for ease of analysis.

### Data analysis

This study was underpinned by Qualitative Descriptive methodology [12] to conduct a thematic analysis of participant descriptions of the processes of knowledge translation undertaken at their respective organisations. This methodology allowed the researchers to move easily into and out of the data, and did not necessitate a highly abstract rendering or theorising of the data [12]. Rather, the analysis entailed “*the presentation of the facts of the case in everyday language*” ([12], p. 336). Qualitative description is considered particularly useful when exploring questions of relevance to policymakers and practitioners [12].

All data analysis was undertaken by the lead author, with review input from two further researchers (PW and RW) experienced in qualitative research, and particularly analysis of interview data, who assisted with developing the terminology around emerging themes. Analysis identified emergent themes and common considerations and processes between organisations. Participants provided accounts that ranged in depth, both within and across interviews. To ensure that the participants were given ample opportunity to provide the most accurate descriptions of process that they could, with the information that they had available at the time, each participant was sent the interview questions in advance. Further, participants were provided the opportunity after transcription to check and amend their accounts.

## Results

This section describes the three themes that emerged from the data, namely (1) identifying a need for knowledge translation, (2) developing and disseminating resources, and (3) barriers and enablers to knowledge translation. The themes presented in Table 1 are an overview of the general practices discussed by the participants from the NoGAPS organisations, and summarises the types of knowledge translation decisions and activities discussed by participants.

**Table 1:** A general overview of the knowledge translation considerations and processes undertaken by the NoGAPS organisations (NoGAPS: National Guidance for Australian Football Partnerships and Safety)

Theme		Considerations	Processes
1	Identifying a need for knowledge translation	Identification of issues	(a) Monitoring of research, (b) Monitoring of sport, (c) Monitoring of media, (d) Government, (e) Collaboration, (f) Organisational goals
2	Developing and disseminating resources	Development of resources	(a) Commission of research to underpin resources, (b) Commission of the production of resources, (c) In-house development of resources, (d) Updating or review of existing resources
		Distribution pathways	(a) Direct, (b) Indirect
3	Barriers and enablers to knowledge translation	Barriers	(a) Format, (b) Framing: Injury prevention, health promotion and/or performance enhancement, (c) No clear strategy, (d) Reach/uptake/impact or justification of resources
		Enablers	(a) Framing: Injury prevention, health promotion and/or performance enhancement, (b) Awareness raising of injury prevention or safety issues

### Theme 1: Identifying a need for knowledge translation

Participants discussed procedures used to identify sports injury prevention and safety promotion issues as the focus of new resources, including monitoring of research, monitoring of sport itself or monitoring of the media for new issues arising:

“*…one process is through our research and observation processes. The second thing is if we see a high incidence of things or a change of incidence of things we’re always looking for that. Obviously things that are important topics in the community or the media*” [Representative 3]

“*Certainly more recently, the issues were identified simply by keeping monitoring the research both in terms of the information that flows from our research partners as well as via various research newsletters and other sources, particularly sources on the web such as The Conversation, or even through various media articles and occasional searches through Google Scholar to see what is coming out*” [Representative 1b]

“*The two basic premises are where* [organisation] *identifies a need because of some latest research or it’s an area we’ve identified there is no information and that may just be because we’ve been proactive and had a look or it may be because we’ve had a number of enquiries about a particular topic*” [Representative 5]

Internal organisational goals or collaborative goals between and across NoGAPS organisations were also discussed as sources of initial development decisions:

“*…it might be that an organisation like* [organisation] *comes to us and says, we want information on this made available for community sport, can you put something together?*” [Representative 5]

“*…what we do is consult broadly working with our key stakeholders and funded* [sic] *partners, as well as our specific program experts, campaigns and communications and marketing team across the organisation. Where we’re talking about sport safety resources we would obviously consult with* [organisation] *to know if it is already in existence or was going to add value, as well as speaking with the sports sector that are actually the end-users…*” [Representative 2]

Government agendas were also mentioned as a starting point for development of new resources:

“*In the past there’s been a cross-government committee…and more recently we had a – the government set up a* [committee] *– and the advice from those bodies are very important in getting approval for research and projects*” [Representative 1a]

### Theme 2: Developing and disseminating resources

For all the participating NoGAPS organisations, the process of development of new injury prevention and safety promotion resources began with the commissioning of research. Alternatively, commission of resources could arise from different sources such as consultation with sporting bodies or scientific committees. The NoGAPS organisations typically responded as their organisational capabilities allowed:

“*So if there was a gap that wasn’t being looked at, then it might be around some data that we need to actually get to, then we would actually source that out and then work with the appropriate partners, internal and external, to strategise to get that happening. So there’s a bit of analysis first and research and program development as required to create practical resources*” [Representative 2]

All participants described how in-house development processes within organisations were determined in consultation with scientific committees, research boards or external researchers:

“*…largely just to work with* [organisation] *with our* [programme] *and commission something specific. If it doesn’t require some research it can just be produced based on current information and expert advice and often the expert advice would come from the* [organisation] *members themselves. I think they tend to have a national alignment with communities or expert panels that can provide that input*” [Representative 1b]

“*At some points in the past there was a scientific committee that would write policies and documents. That stopped being used as much. It’s now just coming back in. In the meantime, it’s been a matter of identifying what it is that has to be written and then identifying the best writer, whether it’s something that can be actually – its best practice doesn’t mean lit review, doesn’t need that in-depth research so it doesn’t need a researcher to write it. It might be the national media manager can manage it. Then we get an expert to look at it*” [Representative 5]

“*What we do is because of our – the knowledge we have within our team of sport and the sport club environment at a local level and at other levels but particularly this is aimed at the local level this one. We come up with…we can populate the criteria on a program like that. We then – and we’ve had a lot of meetings with* [organisation] *and gone* [sic] *‘is this criteria applicable’, can we tweak it? And it’s been tweaked along, between us and* [organisation] *we’ve tweaked it to a point. Then from there we release it to say the* [organisation]*…and get feedback. So it has gone through a feedback process*” [Representative 4]

Another factor that the participants discussed was the dissemination of resources. The dissemination pathways that these organisations used were categorised as either direct or indirect. Direct pathways consisted of directly working with state sporting organisations, regional sporting assemblies, or local sporting bodies to provide them with the resources that were deemed necessary to their context and settings, or by reacting to requests from the sporting bodies themselves for resources on specific sport safety issues:

“*…we have direct communications which go through our state bodies to leagues and clubs or in some cases regions. So the states push out that information…In terms of within the states place orders for the* [injury] *resources for their community…*” [Representative 3]

“*The two channels that we use is one is direct or via the sport, there are some things that we go direct to clubs via email and there’s 4500* [sport] *clubs that we have to communicate with, 2500* [sport] *clubs and so the communication is not easy. It’s not always effective but going direct via email at least we know that it gets there*” [Representative 4]

“*…it’s distribution via the sport network. So if it’s a* [sport] *factsheet we’d be sending it to* [sport] *and asking them to promote it through their networks. If it fits with something that a sport was doing, we would send it to the organisation and ask them to promote it through their links*” [Representative 5]

“*Sports come into us identifying the need and that could be a state sporting association, regional sports assembly, association or a club. Organisations can make contact with us and may not even be a funded organisation*, *e.g. We need this. How do we use our defib* [sic]*. They need further guidance*” [Representative 2]

Indirect pathways to dissemination included events or promotions of new resources, via the media, social media and websites, newsletters or apps:

“*…the distribution of something like the app is pretty easy, you put it on the iStore or Google Play and done, it’s effectively – well you can say that’s been distributed*” [Representative 4]

“*We would do a media release that the national media manager does on behalf of us. So then that goes to media channels*” [Representative 5]

“*Social media, campaigns and innovation are new areas for* [organisation] *to work in, providing more opportunities for brand awareness and reaching target audiences*” [Representative 2]

“*There’s a* [programme] *e-news that goes out monthly. So any new resource is highlighted with a link to the resource that’s posted on to the* [programme] *website. It would be put as a news item on the* [organisation] *website*” [Representative 5]

### Theme 3: Barriers and enablers to knowledge translation

A major barrier identified by all participants in the dissemination of resources to community sport was the format in which they received the knowledge from researchers:

“*I would just say there’s a little bit of a gap between research in its purist form versus reviews of research and putting together resources. We have a gap, and by a gap I mean often when we commission research, the end reports and the products are written for researchers by researchers and they’re not really translated well into a product that* [organisation] *can digest and use immediately. It takes on a lot of translation and then some further work before we get to a point where it’s something that we can actually adopt quickly or implement or recommend that other groups pick up and implement*” [Representative 1b]

“*Well it depends on the audience that we want those resources to go to. They will sit with our branding strategy that we’re working with in* [organisation]*, but it’s important to make sure what they’re called and who they’re going to is clear. Is this for the sports sector? Who is it targeted at? That will then guide how we name it and how we work with it and what is its intended outcome*” [Representative 2]

A secondary barrier across these organisations was the confusion between injury prevention, health promotion and performance enhancement. Depending on the overarching aim of the organisation, the need to frame and target resources was different. Framing injury prevention resources as a means to health promotion or performance enhancement was generally considered by these participants to be an important enabler for this group of organisations:

“*So it’s probably one of our priorities … how to get the information in a form that people will pick that up and want to pick it up and know how to use it, as opposed to messages about how risky particular activities are. It’s more about well this is how to improve your performance and at the same time reduce your injury risk, but that’s yet to really take place*” [Representative 1b]

Another barrier discussed by participants was the need to raise awareness that sports injuries are not inevitable, and are thus preventable:

“*Our challenge is to make the most of that situation and keep that positive perception that something could be done around sports injuries alive…Because I think it’s very easy for people just to go back to well nothing can be done about this. It’s too hard. Let’s just not do anything about it. So from my point of view that’s our big challenge at the moment*” [Representative 1a]

This resonated with other participants’ perceptions of the framing divide between sports injury prevention, health promotion and performance enhancement – but as an enabler:

“*Under that is the tackling barriers for participation and that includes the sports injury prevention. So it’s within that area we are supporting…While it’s not explicit in our action agenda (you don’t read sports safety, sports injury prevention) the staff know it’s part of our key work and it’s certainly still a priority…*” [Representative 2]

One participant commented that a major barrier to the development process was the lack of a clear strategy:

“*I don’t think we have a strong forward research strategy from managing the program point of view that would be easier, but then that needs to be tempered with the need to respond to issues as they arise*” [Representative 1a]

This linked with the overarching perceptions of the participants that there was a pressing need to address the assessment of reach or uptake of resources, and to determine their impact. The participants expressed that justification of their research translation role is an important step, and potential enabler, in future funding and allocation of more work in this area:

“*The difficulty on getting good data and all that sort of stuff means it’s very difficult doing or showing that you’ve made an impact, apart from people’s perceptions and so we’re really stuck with – that’s a very hard area to work with, but we’ve just got to work with that, that people at least perceive that doing something is going to have a positive outcome. That’s enough to maintain impetus*” [Representative 1a]

“*…and that will help how we build that program in the future and this is something that’s seen as a very good opportunity for capturing more accurate local sporting clubs evidence. So while we do not have specific stated sports injury goals in our strategic documents (except at project level) this* [type of] *evaluation will hopefully provide the clear justification for us to continue this work*” [Representative 2]

The major concern across all the participating organisations was this lack of a current means to readily measure reach or uptake of resources once they had been developed and disseminated:

“*…internally are unaware of the broader effect the resources are having, given we are not on the ground. So we might have put those resources together but found it difficult to do full follow up on effectiveness*” [Representative 2]

“*…while they’re using it we may not get the feedback from them about uptake or comments regarding value*” [Representative 1b]

“*…maybe seek more formal feedback on that stuff ourselves. Because while I see lots of evidence of uptake I don’t know how much it is* [or] *how strong it really is. So from the supply end I think we’ve done pretty much everything we can. From the users’ end I am not sure exactly how much – what the level of uptake is, what percentage that’s information that we need to get more of so that we know that – we’re pretty satisfied with what the resources are and how they’re presented and developed in terms of people’s receipt of information. But we don’t have really strong research-based information at this stage*” [Representative 3]

## Discussion

The larger NoGAPS project sought to address several ‘gaps’, including policy to practice, efficacy to effectiveness and research knowledge to translation [13]. This discussion links the themes identified in this study with the role that intermediary organisations play in increasing the relevancy, accessibility and legitimacy of research knowledge for end-users. These aspects are highlighted and reflected upon, particularly in relation to the participating NoGAPS organisations and their role as intermediaries in translating research findings into useful resources that can support safe sport practices.

### The (ir)relevancy of research knowledge

As theme 1 showed, research did not necessarily inform the identification of issues for which injury prevention and safety promotion resources were needed. The monitoring of research was only one aspect of this activity, along with monitoring other sources such as government priorities and media coverage. These organisations appear to give as much weight to media and the self-reported needs of sporting clubs as to robust research evidence.

The identification of safety issues to be addressed therefore did not appear to follow a formal process in any of the participating organisations. This ad-hoc approach to the identification of issues that warrant new or updated injury prevention and safety promotion resources suggests a reactive, rather than proactive approach. In the absence of researchers making research knowledge actionable, increasing the ‘relevancy’ of research knowledge by addressing the timeliness, salience and actionability of the format in which the knowledge produced is presented is an important intermediary function performed by organisations in general [4], and by these organisations for the sporting context. Future efforts should be directed towards the best ways for making research knowledge actionable by these organisations and others.

### The (in)accessibility of research knowledge

Research findings written by, and for, researchers were generally not considered useful to these organisations when undertaking a knowledge translation role, as theme 2 showed. As a group, they commented on the pipeline lag between research and practice due to the time and effort needed to understand, translate and format findings into resources suitable for community settings. In their capacity as organisations providing a service to end-users, it remained imperative that resources were not provided to them in the form of ‘pure research’ but rather as easy-to-digest practical resources that could be readily used and easily understood by those developing the resources, and by end-users at community sport clubs. This is a step that researchers in the field of sports injury prevention and safety promotion traditionally do not undertake [6], and it is likely that they expect organisations such as the NoGAPS partners to assume this role. By addressing the formatting and availability –‘accessibility’ – aspect of knowledge translation, key organisations provide a vital intermediary service to end-users in general [4], and by these intermediary organisations for the sporting context. It is apparent from this study that there is a disconnect between how researchers present their research and the needs of organisations who need to act on it. Going forwards, both groups will need to work together to optimally remove this gap.

The NoGAPS organisations placed a distinct focus on understanding and determining the most effective framing of knowledge via resources for their audience. In other words, they struggled with how best to ‘sell’ these resources and the information contained in them to their audience of end-users. Re-framing or contextualising injury prevention or safety promotion information differently according to overarching organisational objectives – such as health promotion and/or performance enhancement – was thus seen as important. Therefore, whilst these organisations do not necessarily explicitly state safety promotion and injury prevention as overarching goals, they do recognise the need and importance thereof, and thus embed this within their general scope.

### The (il)legitimacy of research knowledge

Theme 3 of this study suggests that the participating organisations generally did not evaluate the reach or ultimate uptake of the resources that they developed and disseminated because they generally did not have the time/resources, staff or skill set to do so. This was perceived as a major barrier to the development and updating of resources in the future, as these organisations have no means of showing the impact of their efforts. As with other intervention outcomes, when reach and impact are not routinely evaluated, a significant barrier to identifying the impact of the work is evident [15], and ultimately the credibility, or legitimacy, of research knowledge is thus undermined [4].

### Limitations

Qualitative description is often characterised as ‘basic’, ‘fundamental’, or ‘surface’; however, it can be, and is, useful and appropriate when exploring issues relevant to ‘real-world’ policy and practice [12].

Only six organisations were included in this research; however, these organisations have previously been recognised as key stakeholders in sports injury prevention and safety promotion in Australia [13, 14]. Therefore, the NoGAPS organisations are considered broadly representative of similar organisations that have briefs/action portfolios relating to sports injury prevention and safety promotion beyond that of the NoGAPS project focus. Participants’ demographic data was not collected, as this study was conceptualised as a sub-study under the inclusion criteria and assumptions of the larger NoGAPS partnership project [14]. Whilst the findings of this qualitative study cannot be generalised to wider populations, it is suggested that the experiences of these participants will resonate with other organisations responsible for interpreting research and developing and disseminating resources to assist in the knowledge translation process. Notwithstanding these limitations, and as described elsewhere [14], there was significant consultation with, and recognition of this research by, the NoGAPS organisations.

## Conclusion

Given the ever-growing literature on the importance of implementation and dissemination science for injury prevention and safety promotion in sports settings [10], the development of frameworks to underpin these issues [8, 9], and the proliferation of resources [11], why do injury prevention and safety promotion outcomes remain challenging?

In this study, participants considered that research knowledge is all too often irrelevant, inaccessible and illegitimate for the purposes of resource development and, ultimately, for use by those at community sports clubs. When research is produced solely by researchers for researchers, the gap between research and practice is perpetuated [16]. Indeed, this has been theorised as contributing to the pipeline process in which research is produced at one end, and practice occurs at the other [16, 17]. These participants suggested that a knowledge translation ‘gap’ does exist, and is consistent with prior understandings in this field [5].

A large body of research on sports injury prevention and safety promotion intervention has focused on implementation strategies (and the implied embedded dissemination strategies); yet, this research is almost wholly focused on the efficacy to effectiveness gap [18]. Research evidence in this regard, as it currently stands, has shown support for the efficacy and clinical relevance of sports injury prevention and safety promotion interventions, namely that they ‘work’ [19]. More recently, support for these interventions has been bolstered by implementation plans, suggesting that effectiveness can too be achieved, and that they ‘work in context’ [20]. Research into sports injury prevention and safety promotion tends to stop short at recognising, and thus researching, the role of industry organisations in the translation of knowledge produced – the research-to-policy and practice gap. This study suggests that there is a need for guidance on measuring and demonstrating outcomes related to the impact of dissemination efforts. It must be noted that key examples where this gap has successfully been bridged do exist, including in the larger NoGAPS project and its successful FootyFirst programme [20]. Further, models such as the non-hierarchical organisational model [21], have been proposed as a means by which constant data and information exchange can be enhanced both across and within end-users.

This study found that key intermediary organisations can, and do, take on a knowledge translation role in order to make research knowledge more relevant (timely, salient, actionable), accessible (formatted and available), and legitimate (credible) for end-users. Indeed, this study echoes a previous finding that “*the greatest barrier to implementation had nothing to do with implementation*” ([22], pp. 223–4), but rather in understanding and influencing the complexity of processes that exist between knowledge and action, in which the participating organisations play an important intermediary role in the process. Recognising and capitalising on the potential intermediary role that these types of organisations play could enhance the influence of research on policy and practice. Thus, such organisations may have an important role to play as key contributors to broad teams tasked with generating injury prevention evidence and ensuring its uptake as an integral part of future research studies, and should be included in the process.

## Additional file

Additional file 1: Appendix A. Interview schedule. (DOCX 16 kb)

## Abbreviations

NoGAPS: National Guidance for Australian Football Partnerships and Safety
