# Supplementary material for: The translation of sports injury prevention and safety promotion knowledge: insights from key intermediary organisations
Source: Health Res Policy Syst. 2017 Mar 28;15:25. doi: 10.1186/s12961-017-0189-5 (PMC5371252; doi:10.1186/s12961-017-0189-5)
Supplement: Additional file 1: — Appendix A. Interview schedule. (DOCX 16 kb) [file 12961_2017_189_MOESM1_ESM.docx]

**THE TRANSLATION OF SPORTS INJURY PREVENTION AND SAFETY PROMOTION KNOWLEDGE: INSIGHTS FROM KEY INTERMEDIARY ORGANISATIONS**

**Additional file 1: Appendix A**

*Interview Schedule*

| What process does [organisation] follow to develop new injury prevention and safety promotion resources? | |
| --- | --- |
|  | How does [organisation] identify new issues for resource development? |
|  | How does [organisation] prioritise new issues for resource development? |
|  | How does [organisation] decide who is involved in the development process? |
|  | How does [organisation] generally develop new content? Is it evidence-based? |
|  | How does [organisation] decide on the description of title of the resource? |
|  | How do you feel the process works? What has worked well / not so well? (successes / failures) |
|  | What are the future plans for the development of new resources? |
|  | Would you do things differently if you could? What would you do differently? |
| How does [organisation] distribute injury prevention and safety promotion resources to community sport clubs? | |
|  | How does [organisation] identify pathways for the distribution of resources to community sports clubs? |
|  | How does [organization] monitor the uptake of resources at community sports clubs? |
|  | Does [organisation] collect feedback about the use of resources at community sports clubs? |
|  | How well do you feel this distribution process works? What has worked well / not so well? (successes / failures) |
|  | What are the future plans for distribution of resources? |
|  | Would you do things differently if you could? What would you do differently? |
